# Rhizobacteria-Mediated Reprogramming of Phytohormone Landscapes for Mitigating Salinity Stress in Plants

**DOI:** 10.3390/ijms27167494

**Published:** 2026-08-21

**Authors:** Arghyadeepa Moharana, Lochan Dhruw, Armita Chakraborty, Preeti Pashwan, Sanjida Sultana Keya, Md. Mezanur Rahman, Archita Singh, Mamta Bhardwaj, Lam-Son Phan Tran, Aarti Gupta

**Affiliations:** 1 Functional Plant Biology Laboratory, Department of Botany, Dr Harisingh Gour Vishwavidyalaya, Sagar MP 470003, India; 2 Center for Biotechnology & Genomics, Texas Tech University, Lubbock, TX 79409, USA; 3 Biosciences Division, Oak Ridge National Laboratory, Oak Ridge, TN 37831, USA; 4 Plant Physiology and Molecular Biology Laboratory, Department of Botany, Dr. Harisingh Gour Vishwavidyalaya, Sagar MP 470003, India; 5 Department of Botany, Hindu Girls College, Maharshi Dayanand University, Sonipat HR 131001, India; 6 Institute of Genomics for Crop Abiotic Stress Tolerance, Department of Plant and Soil Science, Texas Tech University, Lubbock, TX 79409, USA

**Keywords:** abscisic acid, auxins, brassinosteroids, cytokinin, ethylene, jasmonic acid, plant-growth-promoting bacteria (PGPRs), root architecture, strigolactones

## Abstract

Salinity stress is one of the major stressors that limits yield potential in field crops. Salinity-led imbalances in ionic and water potential, as well as oxidative damage, impair photosynthesis. Plant-growth-promoting rhizobacteria (PGPRs) have been demonstrated to mitigate salinity-stress-induced damage through various mechanisms such as biofilm and exopolysaccharide production, modulation of plant root architecture or molecular signaling involving modulation of sodium/potassium efflux transporters. PGPRs are known to induce biosynthesis and signaling of various phytohormones in plants. PGPR-derived phytohormones can in turn regulate molecular signaling involved in maintaining ion fluxes, preventing salinity-induced senescence, and reinforcing plant root architecture, thereby maintaining plant growth and development under saline conditions. In this review, we provide comprehensive advances on how PGPRs modulate and integrate biosynthesis and/or signaling of various phytohormones, such as auxins, cytokinins, gibberellin, ethylene, abscisic acid, salicylic acid, jasmonates, brassinosteroids and strigolactones, to reshape plant architecture, physiological and biochemical responses in plants under salinity. We integrate molecular evidence with morpho-physiological studies and propose a phytohormone-centric framework to select strains that optimize growth, ion homeostasis and plant stress resilience under salinity.

## 1. Introduction

Soil salinity is a major environmental constraint affecting nearly 20% of irrigated agricultural lands globally, which is predicted to increase further due to climate change, poor irrigation practices, and seawater intrusion [1,2]. Increased soil salinity leads to osmotic stress, ion toxicity (sodium/chloride, Na^+^/Cl^−^), limited nutrient uptake and oxidative stress, which all together limit plant growth and yield potential [1,2,3,4]. Plants are equipped with various physiological, biochemical, and molecular mechanisms such as osmolyte accumulation, ion compartmentalization and efflux, and activation of stress-responsive genes to cope with salinity [2,3]. However, most high-yield crops bred for optimal growth in non-stress conditions are incapable of mounting adaptive responses to salinity when cultivated under intensive agricultural practices [2].

In recent years, rhizosphere microbiota has gained attention as a crucial player in plant stress biology [5,6,7]. Plant-growth-promoting rhizobacteria (PGPRs) represent a diverse group of beneficial rhizosphere microbes that colonize either root surfaces or intracellular spaces in the roots, and enhance plant growth and stress tolerance through multiple direct and indirect mechanisms [8,9,10]. PGPRs secrete phytohormones, facilitate nutrient acquisition in plants, enhance antioxidant activity, and trigger induced systemic tolerance (IST) in host plants [8,10]. PGPRs also modulate phytohormone metabolism and associated signaling in host plants, and release volatile and exopolysaccharide signals that regulate plant responses under salinity [11].

Phytohormones such as abscisic acid (ABA), auxins (AUXs), brassinosteroids (BRs), cytokinins (CKs), ethylene (ET), gibberellins (GAs), jasmonic acid (JA), salicylic acid (SA) and strigolactones (SLs) play pivotal roles in orchestrating plant growth and regulating responses to environmental stressors [12]. Under salinity stress, the balance of these hormones is disrupted, leading to impaired growth and reduced stress tolerance [12,13,14,15,16,17]. PGPRs either secrete phytohormones or alter levels and signaling of endogenous phytohormones in plants, thereby helping plants optimize resource allocation, root development, stomatal regulation, and antioxidant and osmolyte accumulation under salinity [10,11,12,18,19,20,21,22,23,24,25,26,27,28]. In this review, we comprehend current knowledge on the influence of PGPRs on phytohormone homeostasis and signaling under salinity stress, discuss the underlying mechanisms and highlight emerging prospects for leveraging PGPRs in salinized fields.

## 2. Morpho-Physiological and Molecular Plant Responses to Salinity Stress

**Figure 1:**
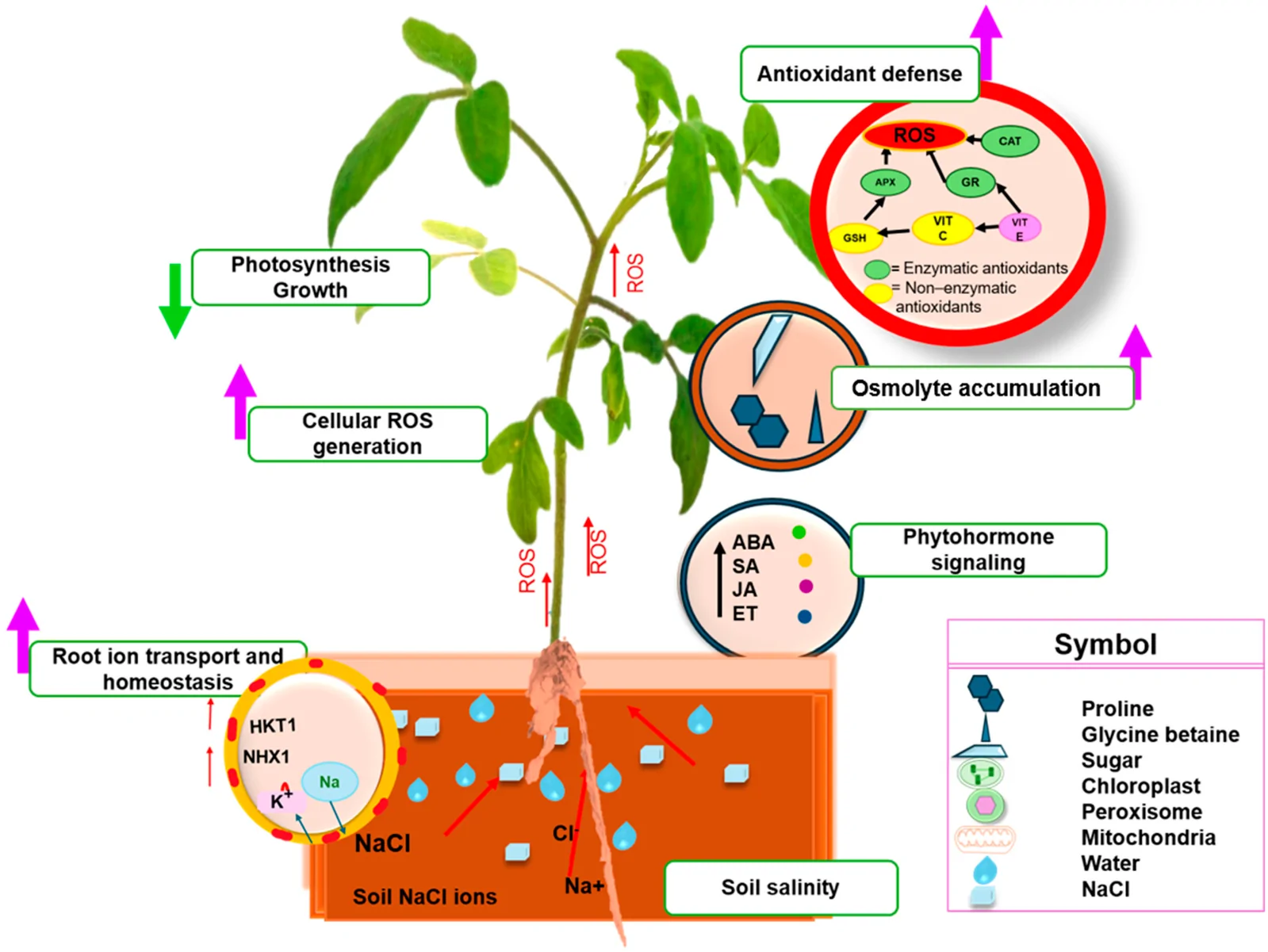
Demonstration of various impacts of soil salinity on plants and plant counter-defense mechanisms. Increased soil salinity (indicated by red arrows) leads to excessive accumulation of Na^+^ and Cl^−^ ions around the root zone, causing osmotic stress and ionic toxicity that initiate stress signaling in plants. Salinity-induced damage includes reduced chlorophyll content, decreased photosynthetic efficiency, and partial chlorosis in leaves. At the root level, increasing Na^+^ competition reduces K^+^ uptake, resulting in Na^+^/K^+^ imbalance. To counter this, plants maintain ion homeostasis through selective ion transport mechanisms involving the High-Affinity Potassium Transporter (HKT1) and Na^+^/H^+^ exchanger (NHX1)-mediated Na^+^ exclusion and compartmentalization. Salinity stress enhances cellular generation of reactive oxygen species (ROS), including hydrogen peroxide (H_2_O_2_), superoxide (O^—2^), hydroxyl radicals (OH^—^), leading to cellular damage. Accumulation of different osmolytes (proline, sugars, glycine betaine) contributes to osmotic adjustment and cellular protection in plants under salinity stress. Furthermore, to neutralize oxidative damage, plants activate antioxidant defense systems comprising enzymatic defense systems such as SUPEROXIDE DISMUTASE (SOD), CATALASE (CAT), and ASCORBATE PEROXIDASE (APX), along with non-enzymatic antioxidants including glutathione (GSH) and Vitamin C (Vit C). Phytohormone signaling mediated by abscisic acid (ABA), salicylic acid (SA), jasmonic acid (JA) and ethylene (ET) integrates salinity-induced stress signals to coordinate ion homeostasis, antioxidant defense, osmotic adjustment, and growth regulation. Purple-colored arrows indicate upregulated processes, and green-colored arrows indicate downregulated processes.

High salinity is mainly reflected by the buildup of Na^+^ in the soil [29]. Salinity adversely affects plant growth and germination, with most crops showing reduced biomass and growth at sodium chloride (NaCl) concentrations ranging from 100 to 400 mM [30]. Salinity-induced reductions in root and shoot length, root architecture, shoot diameter, tiller and leaf number serve as key morphological markers in affected plants [30]. Plants exposed to soil salinity experience a state of dual stress that includes osmotic stress and ion toxicity [1,3,17,29] (Figure 1). Ionic stress caused by high levels of Na^+^ and Cl^−^ ions disrupts cellular homeostasis and enzyme activities, while osmotic stress reduces water uptake in salt-stressed plants [1,17,29] (Figure 1). Salinity also leads to oxidative stress and reduced photosynthetic efficiency, which, together with stunted root growth, leads to nutrient deficiency associated with reduced potassium (K^+^) uptake [31] (Figure 1).

Plants, however, counter the effects of salinity through multiple adaptive strategies [29,31]. One of the primary plant responses involves ion homeostasis by maintaining Na^+^/K^+^ balance via selective ion transporters, such as HIGH-AFFINITY POTASSIUM TRANSPORTER 1 (HKT1) and Na^+^/H^+^ EXCHANGER 1 (NHX1), which contribute to Na^+^ detoxification by retrieving/diverting Na^+^ from the xylem stream to protect the shoots from Na^+^ toxicity [31] (Figure 1). Plants rapidly perceive salt stress through changes in Na^+^ concentration and osmotic pressure, leading to instant increases in cytosolic calcium ion (Ca^2+^) levels [32]. The SALT OVERLY SENSITIVE (SOS) pathway (consisting of SOS3–SOS2–SOS1) decodes these calcium signals to maintain ionic homeostasis [32] (Figure 1). In *Arabidopsis thaliana*, osmosensors, such as REDUCED HYPEROSMOLALITY-INDUCED [Ca^2+^]_i_ INCREASE 1 (OSCA1) and MONOCATION INDUCED Ca^2+^ INCREASES 1 (MOCA1), detect osmotic and Na^+^-induced calcium signals, while FERONIA and calcium-permeable transporters—ANNEXINs—sense cell wall stress and regulate calcium transients [33,34,35,36]. The Ca^2+^-permeable MECHANOSENSITIVE ION CHANNEL MSCS-LIKE (MSL) and MID1-COMPLEMENTING ACTIVITY (MCA) perceive turgor and structural changes, and hydrogen peroxide (H_2_O_2_) bursts further amplify calcium signaling via a leucine-rich-repeat (LRR) receptor kinase HYDROGEN-PEROXIDE-INDUCED CA^2+^ INCREASES 1 (HPCA1) [32]. Together, these interconnected pathways activate downstream transcription factors and ion transporters that sustain growth and protect plants under salt stress [32,37]. In response to salinity stress, phytohormone homeostasis and signaling are fine-tuned to regulate ion transport, mitigate Na^+^ toxicity and optimize root structure architecture [29] (Figure 1). The accumulation of osmolytes such as proline, glycine betaine, and sugars is one of the key strategies for osmotic adjustments during plant growth under saline conditions (Figure 1). Reactive oxygen species (ROS) are generated primarily in chloroplasts, mitochondria, and peroxisomes during normal cellular metabolism, with their production increasing under salinity stress. Although excessive ROS cause oxidative damage, controlled ROS levels function as important signaling molecules that activate stress-responsive pathways and antioxidant defenses, thereby facilitating plant adaptation to salinity [31,38]. Plants activate enzymatic antioxidants such as SUPEROXIDE DISMUTASE (SOD), CATALASE (CAT) and ASCORBATE PEROXIDASE (APX), along with non-enzymatic antioxidants such as flavonoids, which together constitute the plant’s antioxidant defense system and neutralize excessive salinity-induced ROS [31,38] (Figure 1).

## 3. Role of PGPRs in the Mitigation of Salinity Stress

**Figure 2:**
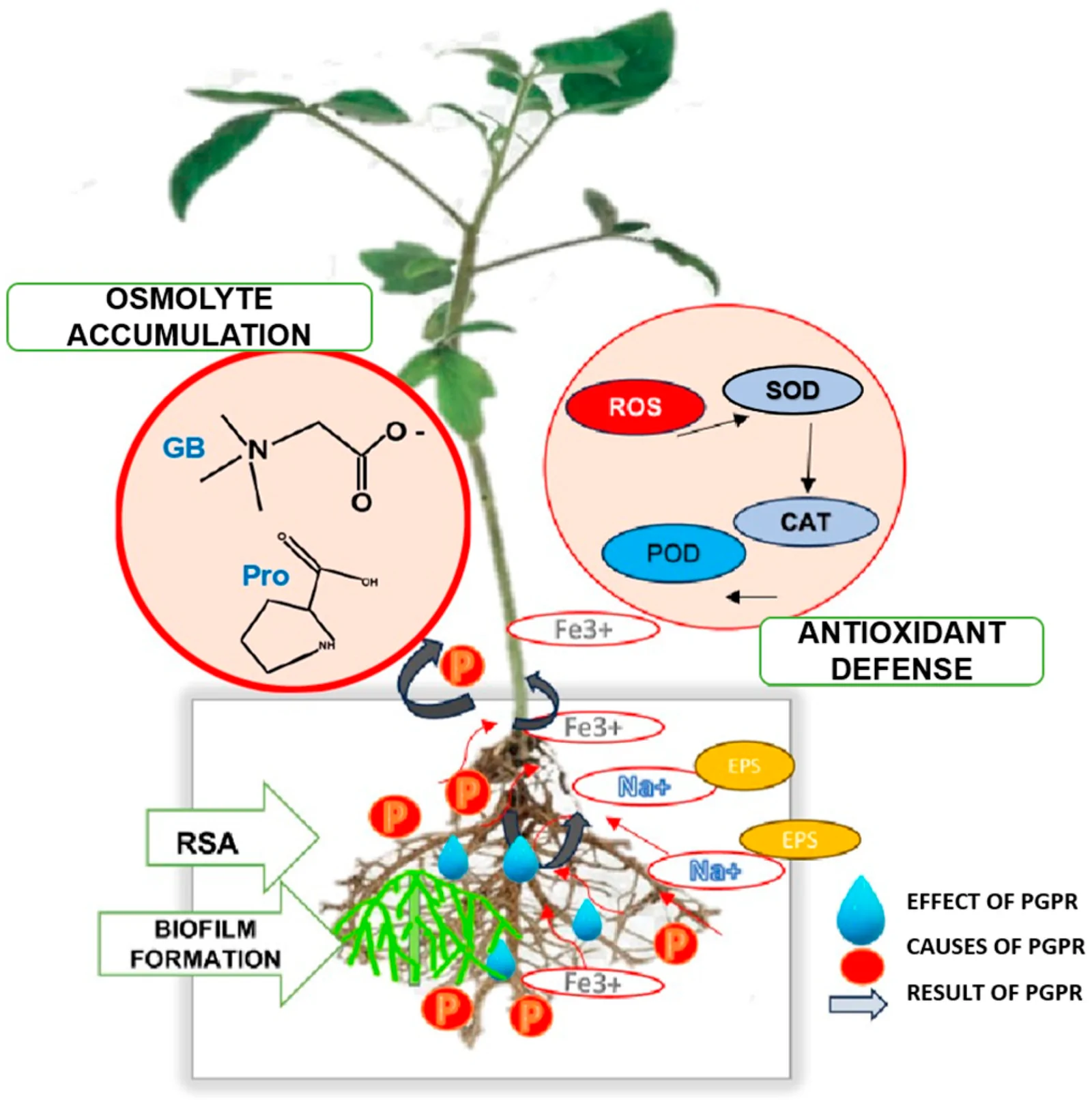
Influence of plant-growth-promoting rhizobacteria (PGPRs) in the mitigation of salinity stress in plants. Excessive accumulation of Na^+^ and Cl^−^ ions causes osmotic stress in salinity-exposed plants, leading to disruption of nutrient homeostasis, decreased iron uptake, chlorophyll degradation and enhanced oxidative stress. These physiological and biochemical mechanisms lead to growth retardation and reduced plant survival. However, inoculation with PGPRs belonging to taxa such as *Azospirillum*, *Bacillus*, *Pseudomonas*, *Enterobacter*, *Exiguobacterium*, and *Rhizobium* alleviates the detrimental effects of salinity stress. PGPRs improve root architecture and enhance nutrient uptake, reduce Na^+^ toxicity by maintaining ionic balance and improve overall plant health. PGPR inoculation also leads to increased accumulation of antioxidants, such as SUPEROXIDE DISMUTASE (SOD), CATALASE (CAT) and PEROXIDASE (POD), and osmolytes, which decrease reactive oxygen species (ROS)-induced oxidative damage and osmotic stress. These effects increase plant survival and improve yield under saline conditions. PGPRs cause biofilm formation, exopolysaccharide (EPS) secretion, and siderophore production, which in turn (effects of PGPR) reduce Na^+^ toxicity and improve nutrient uptake, leading to (result of the PGPR) reinforcement of root structure architecture (RSA) and overall plant growth under salinity. GB, glycine betaine; Pro, proline.

Different taxa of PGPRs, such as *Azospirillum*, *Arthrobacter*, *Bacillus*, *Burkholderia, Pseudomonas*, *Enterobacter*, *Kosakonia*, *Kocuria*, and *Rhizobium,* have shown positive roles in mitigating salt stress in various plant species [8,11,19,23,24,25,26,27,28,37,39,40,41,42,43,44,45]. These PGPRs have been documented to mitigate salinity stress through multiple biochemical and molecular mechanisms [20,46,47,48,49] (Figure 2). For example, inoculation with *P. fluorescens*, *B. pumilus*, and *Exiguobacterium aurantiacum* improved root growth, increased levels of the osmolyte proline, enhanced chlorophyll content, and increased root calcium uptake and yield in *Triticum aestivum* (wheat) under salinity stress [48]. Inoculation with the *B. subtilis* strain IS1 and the *B. amyloliquifaciens* strain IS6 improved root growth and enhanced the contents of chlorophyll, carotenoids and total phenolics in *Solanum lycopersicum* (tomato), leading to improved salt tolerance [46] (Figure 2). Inoculation of the *B. subtilis* strain GB03 enhances salinity tolerance in *A. thaliana* by regulating the expression of an ion transporter gene, *HKT1,* differently in roots and shoots. *B. subtilis* GB03 inoculation reduces *HKT1* expression in roots but increases it in shoots, which lowers Na^+^ accumulation in salinity-stressed plants [50]. However, this effect was not seen in *A. thaliana hkt1* mutants, which showed poor growth and high Na^+^ levels under salinity stress [50].

One of the direct mechanisms by which PGPRs protect plants from salinity involves the production of high-molecular-weight sugar polymers, namely, exopolysaccharide (EPS) [49]. Evidently, EPS has been shown to form biofilms on root surfaces, bind Na^+^ to reduce salt ion toxicity and help improve soil aggregation [49] (Figure 2). Co-inoculation of *A. thaliana* with the halotolerant *Kushneria* isolates *K. konosiri* and *K. marisflavi* enriched *Bacillus* species in the rhizosphere, increased EPS production and biofilm formation, and reduced intracellular Na^+^ and reactive oxygen species levels, thereby alleviating salinity stress [51]. Salinity-induced restriction of plant root growth leads to poor nutrient uptake and absorption, hence disrupting plant growth (Figure 2). PGPRs can also mitigate salinity stress in plants through some indirect mechanisms, namely, enhancing nutrient uptake and increasing plant growth [20,47,51]. For example, most PGPRs produce low-molecular-weight secondary metabolites, namely, siderophores that chelate and transport iron, enhancing iron (ferric ions, Fe^3+^) availability to host plants. In addition, PGPRs also solubilize phosphate [20,47,51] (Figure 2).

## 4. Rhizobacteria-Mediated Phytohormone Regulation

A growing number of transcriptomic and metabolomic studies reveal that PGPRs reshape hormone biosynthesis, signaling, and crosstalk hubs [18,21,52,53]. PGPR-mediated production of phytohormones such as AUXs, CKs, GAs and SA enhances root architecture and growth and helps increase tolerance to saline conditions in various plant species [18,21,52,53,54]. An invariable mechanism exhibited by most PGPRs is the 1-AMINO CYCLOPROPANE 1-CARBOXYLIC ACID (ACC) DEAMINASE (ACCD) activity that reduces plant ET levels, thereby preventing premature senescence under salinity [21].

### 4.1. ABA

**Figure 3:**
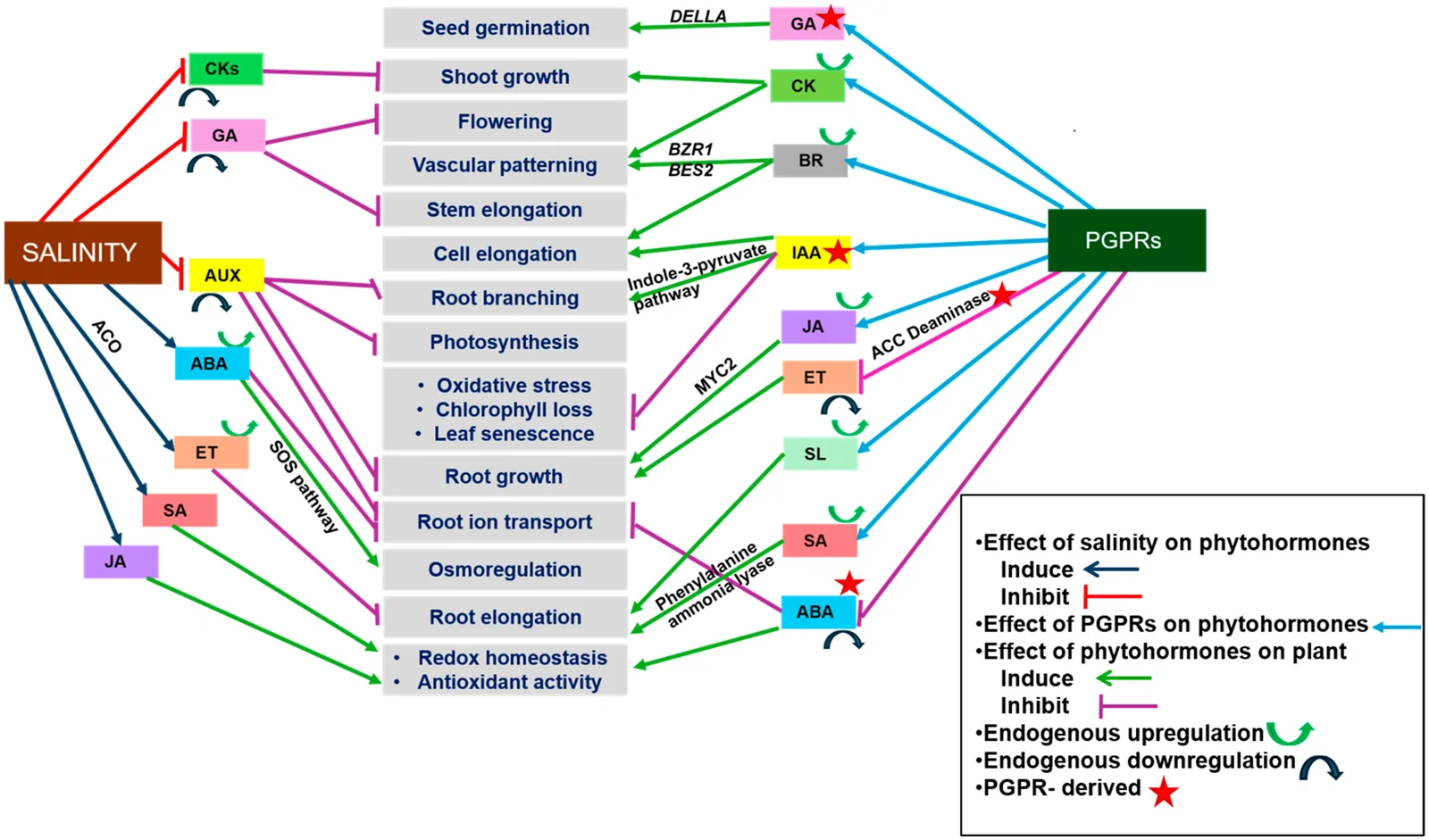
Scheme showing plant-growth-promoting rhizobacteria (PGPR)-mediated phytohormone action/signaling counteracting salinity-induced phytohormone action/signaling in plants. Under salinity stress, plants exhibit increased production of reactive oxygen species (ROS), leading to oxidative damage and hormonal imbalance. Salinity reduces endogenous cytokinin (CK) levels, thereby inhibiting shoot growth. Salinity also leads to downregulated gibberellin (GA) responses, causing reduced flowering, decreased shoot elongation, and inhibited root growth. Salinity invokes elevated activities of ethylene (ET) biosynthetic enzymes 1-Amino cyclopropare 1-carbobylate (ACC) OXIDASE (ACO) and increased ET production. It also elevates abscisic acid (ABA) levels, which induces the salt overly sensitive (SOS) pathway and promotes osmolyte accumulation while decreasing root ion transport. Salinity also decreases auxin (AUX) levels, decreases root ion transport, causing inhibition of root elongation and impaired root development. PGPRs mitigate these adverse effects by restoring hormonal homeostasis and enhancing salinity tolerance in inoculated plants. PGPRs stimulate endogenous CK accumulation, promoting shoot development. They secrete indole-3-acetic acid (IAA) biosynthesis and induces host AUX signaling genes. This results in enhanced cell elongation, increased root branching, delayed leaf senescence, reduced chlorophyll degradation, and alleviation of oxidative stress. PGPRs also enhance brassinosteroid (BR) signaling via upregulation of *BRASSINAZOLE-RESISTANT 1* (*BZR1*) and *BR-INSENSITIVE 1 EMS SUPPRESSOR 2* (*BES2*) genes, promoting cell elongation and vascular differentiation. Furthermore, PGPRs activate GA signaling by relieving DELLA-mediated growth repression, thereby improving flowering and root nodule formation. The production of ACC deaminase by PGPRs lowers the cellular ACC pool, reducing salinity-induced ET accumulation and protecting root meristem activity, which promotes root elongation. In addition, PGPRs upregulate salicylic acid (SA), contributing to redox homeostasis and antioxidant defense. They modulate abscisic acid (ABA) signaling by regulating ROS and Ca^2+^ signaling and enhancing *LATE EMBROGENESIS ABUNDANT* (*LEA*) gene expression, leading to osmolyte accumulation and improved salinity tolerance. This modulation is associated with reduced endogenous ABA levels and enhanced IAA and GA activity, ultimately strengthening antioxidant responses under salinity stress. Moreover, PGPRs induce jasmonic acid (JA) signaling possibly through *MYELOCYTOMATOSIS 2* (*MYC2*), stimulating root growth and further enhancing stress resilience.

ABA has a well-established role in maintaining ionic homeostasis and in the accumulation of osmolytes [29,55] (Figure 3, Table 1). Consistent with this role, *Arabidopsis* ABA biosynthesis and signaling mutants are unable to redirect root growth to avoid high Na^+^ concentrations and exhibit increased Na^+^ accumulation in shoots [56]. Although rapid ABA accumulation is essential for initiating adaptive responses to salinity, prolonged or excessive ABA accumulation can inhibit leaf expansion, photosynthesis, and biomass production. Therefore, the dynamic maintenance of ABA homeostasis, rather than sustained ABA accumulation, is critical for balancing stress tolerance with plant growth [57,58]. For instance, inoculation of *Cucumis sativus* (cucumber) rhizosphere with *B. cereus* BC56 led to a 50% reduction in leaf ABA levels and enhanced salt tolerance [42]. Likewise, *Bacillus subtilis* IB-22 and *Pseudomonas mandelii* IB-Ki14 increased ABA accumulation in wheat roots while reducing its levels in leaves, thereby improving root hydraulic conductivity, water uptake, chlorophyll retention, leaf area, and transpiration under salinity [59]. In an ABA-deficient barley mutant, *B. subtilis* IB-22 further increased root ABA by upregulating the ABA biosynthetic gene *9-CIS-EPOXYCAROTENOID DIOXYGENASE 2* (*HvNCED2*) and downregulating the ABA catabolic gene *CYTOCHROME P450, FAMILY 707, SUBFAMILY A, POLYPEPTIDE 1* (*HvCYP707A1*), while maintaining lower shoot ABA, suggesting restricted root-to-shoot ABA transport during salt stress [60]. Collectively, these findings indicate that PGPR enhance salinity tolerance through the spatiotemporal regulation of ABA, maintaining elevated root ABA to support water uptake while preventing excessive ABA accumulation in leaves that could compromise photosynthesis and growth.

### 4.2. AUXs

AUXs regulate root elongation, lateral root initiation, and cell division [52,68,69]. Low levels of endogenous indole-3-acetic acid (IAA, AUX) were recorded in leaves and roots of *A. thaliana* and rice grown in high salt conditions [16,52,70]. Disruption of AUX distribution under salinity is documented as an adaptive growth response for roots to grow away from salinity, which, however, leads to poor root growth [52] (Figure 3, Table 1). PGPR-synthesized IAA stimulates root branching and increases root surface area, enhancing water and nutrient uptake under saline conditions (Figure 3, Table 1). PGPR-derived IAA enhances root development in *Brassica napus* (canola) plants [22]. The primary roots from canola seeds treated with wild-type *P. putida* GR12-2 were significantly longer than the roots from canola seeds treated with the *P. putida* GR12-2 mutant defective in IAA secretion or the roots from uninoculated canola seeds [22]. Inoculation of *Gossypium hirsutum* (cotton) plants with either of the PGPRs, namely, *B. sonorensis*, *B*. *cereus*, *B*. *subtilis*, *B. tequilensis, Brevibacillus* sp. and *B. pumilus,* resulted in longer roots and improved tolerance to salinity, which was attributed to the IAA-producing ability of these PGPRs under saline conditions [23,71].

PGPR-derived IAA reinforces root structure architecture with longer primary roots and increased lateral roots, which in turn improves water and ion foraging and K^+^/Na^+^ selectivity under saline conditions [23]. Inoculation of *A. thaliana* with *Pseudomonas* sp. A-2 caused enhanced IAA production and root growth under saline conditions [72]. The expression levels of genes related to AUX-dependent root development, namely, *AUXIN RESPONSE FACTORs* (*ARFs*), *AMIDASE 1* (*AMI1*), *TRYPTOPHAN AMINOTRANSFERASE OF ARABIDOPSIS 1* (*TAA1*), *YUCCAs* (*YUCs*), *INDOLE-3-BUTYRIC ACID* (*IBA*) *RESPONSEs* (*IBRs*), *TRANSPORTER OF IBA1* (*TOB1*), and *ENOYL-COA HYDRATASE 2* (*ECH2*), were induced in the treated *A. thaliana* plants, consistent with improved root growth under salinity [72] (Figure 3, Table 1). Salt-tolerant *Kosakonia sacchari* MSK1 produced 14% more IAA content in a high-salt (400 mM NaCl) culture medium when compared with the control culture medium [28]. *Vigna radiata* (mung bean) plants inoculated with *K. sacchari* MSK1 exhibited improved salt tolerance and yield when compared with uninoculated plants under salinity [28].

### 4.3. BRs

BRs are steroid hormones that mitigate the damaging effects of salt stress. Exogenous treatment of 2,4-epibrassinolide (EBR) on *Capsicum annuum* (chili pepper) leaves under salinity increased accumulation of sugars and glycine betaine, which in turn ameliorated ROS and malondialdehyde (MDA) accumulation [73]. A study by [74] highlights the role of PGPR-mediated modulation of BR signaling in improving plant tolerance to salinity (Table 2). Inoculation of *A. thaliana* with *B. endophyticus* J13 resulted in upregulation of the genes encoding positive regulators of BR signaling, BRASSINAZOLE-RESISTANT 1 (BZR1) and BR-INSENSITIVE 1 (BRI1, a receptor for BRs) EMS SUPPRESSOR 2 (BES2), and downregulation of a gene encoding a negative regulator of BR signaling, BRASSINOSTEROID INSENSITIVE 2 (BIN2), and enhanced salinity tolerance [74] (Figure 3). It has been demonstrated that under salt stress, active BIN2 negatively regulates the SOS (Salt Overly Sensitive) pathway by phosphorylating SOS2, thereby limiting SOS1-mediated Na^+^ extrusion [32,75]. Inhibition of BIN2 by BR signaling relieves this repression, allowing the SOS2–SOS3 complex to activate the plasma membrane Na^+^/H^+^ antiporter SOS1, which promotes Na^+^ efflux, reduces cytosolic Na^+^ accumulation, and helps maintain K^+^ retention. Consequently, plants maintain a favorable Na^+^/K^+^ ratio, minimizing ionic toxicity and preserving cellular homeostasis under saline conditions [32,75].

### 4.4. CKs

CKs are known to play key roles in plant stress responses by regulating cell division, leaf expansion, and delaying senescence [79,80]. Under salinity stress, endogenous CK levels have been shown to decline, leading to growth retardation [80] (Figure 3). Inoculation of tomato plants with *B. velezensis* HR6-1 enhances salt tolerance, which is associated with increased endogenous CK content and quenching of salinity-induced ROS burst [53]. This PGPR-induced improvement in salt tolerance in tomato plants was inhibited upon treatment with a CK synthesis inhibitor, namely, lovastatin, indicating the key role of CKs in mediating this effect [53] (Table 2). Similarly, inoculation of durum wheat (*T. durum*) plants with CK-producing *B. subtilis* IB-22 increased the concentration of CKs in salt-stressed plants [41]. Inoculation with *B. subtilis* maintained endogenous ion fluxes in these plants by promoting the formation of Casparian strips in the endodermis, suggesting a potential link between CK accumulation and anatomical adaptations [41]. However, this relationship requires further investigation.

### 4.5. ET

High salinity has been shown to elevate the activities of ET biosynthetic enzymes ACC OXIDASE (ACO) and increase ET production in *Zea mays* (maize) roots [81]. Salt-induced ET suppresses the activities of plasma membrane H^+^-ATPase and SOS1 proteins, thereby disrupting Na^+^/H^+^ balance and reducing Na^+^ efflux in maize roots [81]. In rice, inoculation with *Bacillus subtilis* (NBRI 28B and NBRI 33N), *B. safensis* (NBRI 12M), and *Jeotgalicoccus huakuii* (NBRI 13E) enhanced salinity tolerance by modulating ET homeostasis. PGPR reduced salt-induced ethylene accumulation through strain-specific regulation of ACC content and ACC synthase activity, while simultaneously improving antioxidant defense, primary metabolism, and plant growth under saline conditions [62] (Table 2). *Phaseolus vulgaris* (French bean) inoculated with *Aneurinibacillus aneurinilyticus* and *Paenibacillus* exhibited reductions in salinity-induced ET levels, which were associated with the ACCD activity of these PGPRs [82]. PGPRs possessing the ACCD enzyme degrade ACC, the ET precursor, thereby reducing salt-induced ET. Inoculation of *T. aestivum* with *E. cloacae* improved root growth by producing ACCD and reducing ET accumulation under salinity stress [83]. The production of ACCD by the halotolerant *Burkholderia* sp. MTCC 12259 was highly correlated with increasing NaCl concentrations in the culture medium, where ACCD reached the highest level at 600 mM NaCl [84]. Altogether, PGPRs lower plant ACC pools, dampening ET bursts and promoting root elongation to help plants cope with salinity (Figure 3).

### 4.6. GAs

Reduced GA biosynthesis, leading to low levels of endogenous GAs, is associated with salinity-induced stunted growth [4] (Figure 3, Table 1). PGPRs such as *Acetobacter diazotrophicus*, *Herbaspirillum seropedicae*, *Bacillus* spp., or *Azospirillum* spp. and *Bradyrhizobium japonicum* have been shown to produce ent-kaurene, a precursor to GA [27,85]. These effects can potentially be extended to plants growing under saline conditions. *P. putida* H-2-3 secretes GAs in culture medium, and application of *P. putida* H-2-3 restored plant growth in GA-deficient mutant Waito-c rice over control plants. This effect is correlated with the GA-producing effects of *P. putida* [27] (Figure 3, Table 1). Inoculation with GA-producing PGPRs, namely, *Pseudomonas frederiksbergensis* and *Priestia aryabhattai,* enhanced plant performance under saline conditions. These strains produce multiple bioactive GAs (GA_1_, GA_3_, GA_4_, and GA_7_) and significantly improved seed germination, biomass accumulation, and shoot growth in *Malva verticillata* (mallow) and *Brassica oleracea* (broccoli) exposed to salt stress. The enhanced salinity tolerance has been attributed to GA-mediated stimulation of root elongation, which facilitates improved water and nutrient acquisition under saline conditions [63] (Figure 3, Table 1).

### 4.7. JA

JA regulates plant root growth, defense responses, antioxidant activity and redox homeostasis [12] (Figure 3, Table 1). Inoculation of *A. thaliana* plants with halotolerant *P. putida* PS01 led to upregulated expression of the JA biosynthetic gene *LIPOXYGENASE 2* (*LOX2*) and enhanced plant survival under salinity stress [86]. The downregulation of the stress marker genes *APX2* and *GLYOXALASE 17* (*GLY17*) further suggested reduced stress levels in the inoculated plants [86] (Figure 3, Table 1). *B. amyloliquefaciens* FZB42 volatile organic compounds (VOCs) enhanced salt tolerance in *Arabidopsis* without root colonization by increasing biomass, activating antioxidant enzymes [such as PEROXIDASE (POD), CAT, SOD] and reducing In planta Na^+^ accumulation through upregulation of the expression of *NHX1* and *HKT1* genes. This VOC-mediated tolerance was associated with upregulation of JA biosynthesis and signaling under salt stress [11]. Inoculation with *B. japonicum* IRAT FA3 reduced Na^+^ accumulation in *A. thaliana* and enhanced salt tolerance by priming JA signaling and decreasing ROS levels [44]. Inoculated *A. thaliana* plants exhibited increased shoot biomass, reduced lipid peroxidation, and activation of antioxidant defense-related pathways. Notably, the PGPR-induced salt tolerance was lacking in JA-signaling *jasmonate resistance 1* (*jar1*) and *myelocytomatosis 2* (*myc2*) mutants, demonstrating that functional *JAR1* and *MYC2* are essential for *Bradyrhizobium*-mediated growth enhancement under salt stress [44] (Table 2).

### 4.8. SA

Under salinity, moderate SA accumulation contributes to reactive oxygen species detoxification, maintenance of photosynthetic efficiency, and stabilization of cellular membranes [12] (Figure 3). The combined application of salt-tolerant *P. aeruginosa* and exogenous SA alleviated the adverse effects of salinity in maize by improving growth, biomass, chlorophyll content, relative water content, antioxidant enzyme activities (SOD, CAT, APX), ionic balance, and grain yield compared with untreated salt-stressed plants [87]. PGPR and SA combine to mitigate salinity stress, likely by employing a bi-layered defense combining PGPR-driven Na^+^ binding with SA-activated gene regulation and antioxidant defense under high-salt conditions. The *Bacillus* sp. strain L81 and *Arthrobacter oxidans* strain BB1 markedly enhanced plant performance under salinity by lowering salt-induced mortality by 72.4% and 57.8%, respectively [78]. Both strains also significantly improved plant growth and photosynthetic performance under normal and saline conditions. Mechanistic analyses using SA-deficient *Arabidopsis NahG* plants, the JA-deficient *jar1* mutant, and expression profiling of *PR1* and *PDF1*.2 demonstrated that the enhanced stress tolerance, particularly in BB1-treated plants, was predominantly mediated through activation of the SA-dependent signaling pathway [78].

### 4.9. SLs

SLs are shown to enhance root development, modulate stomatal closure and are regarded as positive regulators of abiotic stress tolerance in plants [40,88]. Exogenous application of SLs led to mitigation of damaging effects of salinity in chili pepper [89]. Plant root exudates characteristic of flavonoid compounds and SLs are known to attract and shape rhizobacterial communities [90]. A combination of PGPR and SL treatment improved membrane stability and enhanced antioxidant defense, leading to improved growth of wheat plants under saline conditions [91]. This combinatorial treatment involving wheat seeds inoculated with halotolerant *B*. *velezensis* UTB96 and leaves from treated seeds sprayed with the SL analog GR24 led to reduced Na^+^ content in leaves and roots [91]. Co-inoculation of *A. thaliana* plants with the PGPR *Stutzerimonas stutzeri* and the green algae *Chlorella vulgaris* led to improved salt tolerance [92]. Transcriptome profiling of these inoculated plants revealed downregulated expression of a gene encoding a negative regulator of SL signaling, namely, SUPPRESSOR OF MORE AXILLARY GROWTH 4 (SMXL4) [92].

### 4.10. PGPRs Orchestrate Phytohormone Crosstalk to Coordinate Salinity Tolerance

Phytohormones function as an interconnected signaling network rather than as independent regulators, and accumulating evidence indicates that PGPR-mediated salinity tolerance is largely orchestrated through the modulation of hormone crosstalk. Numerous PGPR strains simultaneously modulate multiple hormone pathways, thereby reprogramming the hormonal network to coordinate root architecture, ionic homeostasis, photosynthesis, antioxidant defense, and osmotic adjustment under saline conditions. Halotolerant rhizobacteria directly synthesize these phytohormones and the corresponding key regulatory enzymes under saline conditions to counteract toxicity. For example, strains of *P. stutzeri*, *Stenotrophomonas maltophilia*, and *P. putida* isolated from the *Coleus* rhizosphere produced IAA, GA, and CK under saline conditions [93]. Likewise, the levels of IAA and ACCD produced by the *Kosakonia sacchari* strain *MSK1* increased with rising NaCl concentrations, up to 400 mM [28]. Similarly, the halotolerant strains *B. haynesii* SFO145, *Salinicola halophilus* SFO075, and *Staphylococcus petrasii* SFO132 secreted IAA and ACCD and improved maize growth under salt stress [25]. *B. amyloliquefaciens* H-2-5 has demonstrated broad-spectrum growth-promoting efficacy across Chinese cabbage, radish, tomato, and mustard plants.

Furthermore, PGPR inoculation rewires signaling networks. Rather than uniformly suppressing or enhancing individual hormones, PGPRs reshape the relative abundance of multiple phytohormones to balance stress tolerance with continued growth, as seen when *B. endophyticus* J13 confers salt tolerance in *A. thaliana* by upregulating BR signaling along with increasing the expression of the plant *ACC SYNTHASE* gene but reducing levels of the polyamine putrescine [74] (Figure 3). One of the prominent traits of PGPR-driven crosstalk is downregulation of plant ABA coupled with activation of protective pathways. In soybean, inoculation with *Arthrobacter woluwensis* AK1 reduces endogenous ABA but increases levels of IAA and GAs and the resultant activation of antioxidant defense under salinity [19] (Figure 3, Table 1). Similarly, inoculation of *P. koreensis* MU2 in soybean plants led to enhanced salinity tolerance, which was correlated with enhanced endogenous SA levels but reduced levels of ABA and JA [94]. This hormonal reprogramming is further demonstrated in cucumber plants inoculated with a triple-species consortium of *Burkholderia cepacia* SE4, *Promicromonospora* sp. SE188, and *Acinetobacter calcoaceticus* SE370 [26]. This consortium effectively ameliorates salinity toxicity by downregulating endogenous ABA while significantly increasing protective endogenous SA and GA_4_ levels [26]. These coordinated hormonal shifts ultimately drive critical downstream physiological improvements, resulting in increased plant biomass, enhanced water potential, and decreased electrolyte leakage under severe salt stress [26].

Interestingly, certain PGPRs have been shown to directly secrete ABA into the rhizosphere, which can exert highly localized, positive effects on root system architecture and initial osmotic adaptation [95]. However, simplistic claims regarding an overall reduction in ABA in inoculated plants can be erroneous. Interestingly, certain PGPRs, including *B. amyloliquefaciens* H-2-5, have been shown to directly secrete ABA, which actively suppresses NaCl-induced stress effects during short-term salinity exposure in soybean by elevating endogenous GA_4_, but lowering endogenous concentrations of ABA, SA, JA, and proline [95]. Hence, in the absence of detailed spatiotemporal profiling, the localized hormone accumulation patterns are obscured and fail to capture the precise tissue-specific dynamics required to accurately define PGPR-mediated salt stress responses.

Key mechanisms of hormonal reprogramming involve microbial volatile organic compounds (VOCs) [96]. The microbial VOC (acetoin) produced by *Bacillus amyloliquefaciens* was able to significantly alter morphological characteristics, which led to higher accumulation of total chlorophyll, increased SA levels, and reduced ABA levels in shoots of *Mentha piperita* under salt stress when compared to uninoculated plants [96]. Similarly, exposure to VOCs from the halotolerant *Glutamicibacter halophytocola* KLBMP 5180 promoted growth, enhanced antioxidant defenses, reduced oxidative damage, and maintained ionic homeostasis, resulting in alleviation of salinity effects. Mechanistically, these effects are driven by the accumulation of endogenous AUXs, JA, and ACC, alongside the activation of their respective signaling pathways [66]. However, application of ET and JA inhibitors led to reversal of this salt tolerance, confirming that KLBMP 5180-derived VOCs function primarily by modulating multiple hormone signal transduction pathways [66].

## 5. Conclusions and Future Perspectives

A comparison of various salt mitigation strategies suggests that PGPR inoculation exceeds traditional physical, chemical, and breeding methods in terms of ecological impacts and cost efficiency (Table 3). The direct biological efficacy of this PGPR inoculation lies in the immediate physiological impact on the host crop.

PGPR-mediated phytohormonal modulation offers a promising approach to enhance plant resilience against salinity stress. By fine-tuning endogenous phytohormonal metabolism and signaling and secreting phytohormones in the rhizosphere, PGPRs improve root system architecture, photosynthesis, and antioxidant defense, help in osmotic adjustment and thereby alleviate adverse effects of salinity on plant growth and yield. Future research should focus on a hormone-centric approach in the selection and screening of PGPR strains, enabling precise modulation of phytohormone homeostasis and crosstalk, and rational consortia for climate-resilient agriculture on salt-affected lands. Following the hormone-modulatory capacities of PGPRs, a judiciously designed PGPR mix or consortium can further provide solutions for climate-proof agriculture. However, successfully transitioning these designed PGPR consortia from controlled laboratory experiments to field applications remains a critical bottleneck that requires resolving fundamental mechanistic gaps. To bridge this gap, future studies must move beyond whole-tissue analyses towards high-resolution spatial and temporal regulation.

Furthermore, given that hormone signaling is highly dynamic, where transient pathway activations trigger downstream feedback loops, integrating spatiotemporal hormone profiling with transcriptomics, metabolomics, and bacterial functional genomics across different plant species and salinity regimes will help distinguish strain-specific responses from conserved mechanisms and resolve the current inconsistencies.

## Figures and Tables

**Table 1:** Studies demonstrating modulation of phytohormone levels and/or signaling in plants inoculated with various PGPRs under saline conditions. ABA, abscisic acid; ACCD, 1-amino cyclopropane 1-carboxylic acid deaminase; ET, ethylene; IAA, indole-3-acetic acid; ICA1d, indole-3-carboxaldehyde; JA, jasmonic acid; RSA, root system architecture; RWC, relative water content; SA, salicylic acid. Upward arrows indicate an increase, and downward arrows indicate a decrease in the corresponding parameter.

S. No.	PGPR Strains	Plant Species	Site of Regulation	Hormone	Salinity Tolerance	Effect on Salinity Response	Ref.
1.	*Bacillus subtilis* IB-22	*Triticum durum*	In planta	↓Root ABA ↑Leaf ABA	↑	↓Stomatal conductance ↓Chlorophyll loss ↑Water uptake ↑Root hydraulic conductivity ↑RSA	[59]
2.	*Rhizobium* E20-8	*Zea mays*	Rhizosphere	↑IAA	↑	↑Proline ↑Antioxidant defense ↑Root growth	[61]
3.	*Streptomyces lasalocidi* JCM 3373^T^	*Glycine max*	Rhizosphere	↑ICA1d	↑	↑Proline ↑Antioxidant defense ↑RSA ↑Expression of AUX-transport genes *GmPIN1a, GmPIN2a* and AUX-biosynthesis *GmYUCCA5 GmYUCCA6* genes	[54]
4.	*Bacillus spp.*, *Jeotgalicoccus huakuii*	*Oryza sativa*	In planta	↓ET	↑	↑Plant growth ↑Photosynthesis ↑Gas exchange ↑Antioxidant defense	[62]
5.	*Pseudomonas frederiksbergensis* *Priestia aryabhattai*	*Malva verticillata, Brassica oleracea*	Rhizosphere	↑GAs	↑	↑Seed germination ↑Shoot growth ↑Root growth ↑Biomass	[63]
6.	*Bradyrhizobium japonicum* IRAT FA3	*Arabidopsis thaliana*	In planta	↑JA	↑	↑Seed germination ↑Plant growth ↑Shoot biomass ↓Lipid peroxidation ↓Na^+^ accumulation ↓Shoot and root weight	[44]
7.	*Kocuria rhizophila Y1*	*Zea mays*	Rhizosphere In planta	↑IAA ↓ABA	↑	↑Biomass ↑Seed germination rate ↑Photosynthesis ↑Antioxidant defense ↑RWC ↑Chlorophyll	[64]
8.	*B. haynesii* SFO145, *Salinicola halophilus* SFO075, *Staphylococcus petrasii* SFO132	*Zea mays*	Rhizosphere	↑IAA ↑ACCD	↑	↑Shoot and root growth	[25]
9.	*Burkholderia pyrrocinia* P10	*Arachis hypogea*	Rhizosphere	↑IAA ↑ACCD	↑	↑Fresh weight ↑Root length ↑Root development ↑Chlorophyll	[65]
10.	*Glutamicibacter halophytocola* KLBMP 5180	*Solanum lycopersicum*	In planta	↑IAA ↑ET ↑JA	↑	↑Photosynthesis ↑Antioxidant defense ↑Ion balance	[66]
11.	*Bacillus atrophaeus* WZYH01, *Planococcus soli* WZYH02	*Zea mays*	In planta	↑IAA ↓ABA	↑	↑Biomass ↑Antioxidant defense ↑Proline, soluble sugars, GSH ↑Nutrient acquisition ↓Na^+^ levels ↑Expression levels of *ZmNHX1*, *ZmNHX2*, *ZmHKT*, *ZmDREB2A*, *ZmWRKY58* ↓Expression levels of *ZmNCED*	[45]
12.	*Pseudomonas putida* KT2440	*Citrus macrophylla*	In planta	↓ABA ↓SA	↑	↓Transpiration (E) ↓Stomatal conductance ↓Quantum yield ↓Root chloride ↓Root proline levels ↑Leaf abscission	[67]

**Table 2:** List of phytohormonal mutants involved in PGPR-induced salt tolerance. *cbb1*, *cabbage 1*, a BR deficient mutant; *cre1*, *cytokinin receptor deficient 1*; *eto1*, *ethylene*-*overproducer 1*; *etr1*, *ethylene response 1*; *jar1*, *jasmonate resistant 1*; *lox4*, *lipoxygenase 4*; *myc2*, *myelocytomatosis 2*, a JA signaling mutant.

S. No.	Phytohormonal Pathway	Mutants	PGPR Strains Tested	Response/Result	Ref
1.	BR	*cbb1*	*Alcaligenes faecalis* JBCS1294 (volatiles/VOCs only)	JBCS1294 VOC induces growth promotion and salt tolerance	[76]
2.	CK	*cre1*	*Alcaligenes faecalis* JBCS1294 (volatiles/VOCs only)	JBCS1294 VOC induces growth promotion and salt tolerance	[76]
3.	ET	*etr1-3*	*Bacillus amyloliquefaciens* FZB42	FZB42 induces plant salt tolerance via activating ET/JA signaling	[77]
4.	ET	*eto1*	*Bacillus amyloliquefaciens* FZB42	FZB42 induces plant salt tolerance via activating ET/JA signaling	[77]
5.	JA	*jar1-1*	*Bacillus amyloliquefaciens* FZB42	FZB42 induces plant salt tolerance via activating ET/JA signaling	[77]
6.	JA	*lox4*	*Bacillus amyloliquefaciens* FZB42 (volatiles/VOCs only)	FZB42 volatiles induce the expression of JA synthesis gene	[11]
7.	JA	*myc2*	*Bradyrhizobium japonicum* IRAT FA3	MYC2 is essential for *B. japonicum* to promote growth and confer salt tolerance	[44]
8.	SA	*NahG*	*Arthrobacter oxidans* BB1	BB1 induces salt stress tolerance through activation of SA-dependent signaling pathway	[78]

**Table 3:** An overview of different approaches to mitigate salinity effects in agriculture.

Approach	Cost	Time to Take Effect	Ecological Impact	Agronomic Benefits	Drawbacks	Refs
PGPR inoculation	Low	Days	Restores soil biology	Boosts nutrient uptake, water retention, and ion homeostasis	PGPRs face survival competition in native soil Efficacy of the isolates varies with plant species and cultivation conditions	[97,98]
Leaching, scraping, deep plowing	High cost of heavy machinery	Weeks	Risks groundwater salinity, disrupts soil structure	Lowers salt levels in topsoil	Requires massive volumes of fresh water	[99]
Soil amendment with gypsum, lime or elemental sulfur	Moderate to high cost of bulk material and transport costs	Weeks–months	Risks of soil compaction or crusting	Replaces availability of Na^+^ ions with beneficial Ca^2+^ ions	High continuous material cost Site-specific variability in performance, varies with soil texture, salinity levels, climate, and water regime	[100,101]
Salt-tolerant cultivars generated through breeding or genetic engineering	High cost of research	Years	GMO regulatory hurdles	High yield security in saline environments	Specific to one crop variety Traits can break down	[102]

## Data Availability

No new data were created or analyzed in this study.
